# Hyperbaric oxygen therapy attenuates brain radiation-induced cognitive deficits in rats

**DOI:** 10.7150/ijms.104248

**Published:** 2025-01-01

**Authors:** Sheng-Yow Ho, Chia-Hui Lin, Chien-Cheng Huang, Cheng-Hsien Lin, Mao-Tsun Lin, Ying-Jan Wang, Jui-Ti Ma, Li-Tsun Shieh, Ching-Ping Chang, Hung-Jung Lin

**Affiliations:** 1 Department of Radiation Oncology, Chi Mei Medical Center, Tainan, Taiwan.; 2 Graduate Institute of Medical Sciences, Chang Jung Christian University, Tainan, Taiwan.; 3 Department of Emergency Medicine, Chi Mei Medical Center, Tainan, Taiwan.; 4 Department of Environmental and Occupational Health, College of Medicine, National Cheng Kung University, Tainan, Taiwan.; 5 Department of Emergency Medicine, Kaohsiung Medical University, Kaohsiung 81201, Taiwan.; 6 Department of Medicine, Mackay Medical College, New Taipei City, Taiwan.; 7 Department of Medical Research, Chi Mei Medical Center, Tainan, Taiwan.

**Keywords:** whole brain radiotherapy, hyperbaric oxygen, neurogenesis, lipid peroxidation, microgliosis

## Abstract

Effective therapies for cognitive impairments induced by brain irradiation are currently lacking. This study investigated the therapeutic potential of hyperbaric oxygen therapy (HBOT) for radiation-induced brain injury in a randomized controlled experimental model using adult male Wistar rats. Adult male Wistar rats were divided into four experimental groups: 0 Gy whole brain radiotherapy (WBRT) with normal baric air (NBA) treatment, 0 Gy WBRT with HBOT, 10 Gy WBRT with NBA, and 10 Gy WBRT with HBOT. Behavioral tests and histochemical analyses were conducted four weeks post-WBRT to assess cognitive function, hippocampal microgliosis, apoptosis, and lipid peroxidation. Compared with the rats with 0 Gy WBRT on 28 days, the rats with 10 Gy WBRT on 28 days had significantly higher severity of spatial learning and memory dysfunction and hippocampal microgliosis, newborn neuronal apoptosis, and lipid peroxidation. HBOT significantly prevented and reversed WBRT-induced cognitive deficits, hippocampal microgliosis, newborn neuronal apoptosis, and lipid peroxidation. In addition, HBOT prevented and reversed the increased apoptosis among newborn neural stem cells and neuroblasts caused by 10 Gy WBRT on 7 days. The findings suggest that WBRT disrupts neurogenesis and enhance microgliosis, apoptosis of neuronal progenitors, and lipid peroxidation in the dentate gyrus, potentially leading to cognitive deficits and neuronal death. HBOT may offer a protective effect against these cognitive impairments and their underlying mechanisms in adult male rats following WBRT.

## Introduction

Whole-brain radiotherapy (WBRT) has long been the primary palliative approach for patients with multiple brain metastases 1. However, with the advent of targeted therapies and advanced treatment options, cancer patients with brain metastases are now experiencing extended survival, sometimes living for years after receiving WBRT 2. Although WBRT effectively eliminates cancer cells, it can cause significant cognitive deficits in up to 90% of patients who undergo the treatment 3-7. These cognitive impairments are likely linked to disruptions in hippocampal neurogenesis caused by irradiation 8-12.

In the adult hippocampus, neuroprogenitor cells in the subgranular zone (SGZ) of the dentate gyrus produce new neuroblasts. The majority of these newly formed cells undergo apoptosis within the first 1 to 4 days as they transition from amplifying neuroprogenitors to neuroblasts 13. In the SGZ of adults, microglia, when unchallenged, phagocytose these apoptotic cells, thereby shaping hippocampal neurogenesis 13, 14. The impact of WBRT on adult hippocampal neurogenesis, mainly through apoptosis-related phagocytosis, alterations in microglial morphology, and lipid peroxidation in the hippocampus, remains uncertain.

Microglia perform various functions, such as promoting neurogenesis, phagocytosing apoptotic neurons, supporting synaptic innervation, forming neural circuits, and facilitating myelinogenesis 15. Under normal physiological conditions, they typically exhibit a ramified morphology with small cell bodies and elongated processes 16. However, when the brain is stimulated by irradiation, microglia take on an amoeboid shape, characterized by larger cell bodies, shorter protrusions, increased phagocytic activity, and the release of various pro-inflammatory mediators and reactive oxygen species (ROS) 17. Persistent activation of microglia can result in chronic neuroinflammation and cognitive deficits, particularly following WBRT 18, 19. In WBRT, the disruption of the delicate balance between reactive oxygen/nitrogen species and antioxidants within cells results in lipid peroxidation, protein degradation, and DNA damage 20.

Hyperbaric oxygen therapy (HBOT) involves delivering pure oxygen at pressures greater than atmospheric levels within a controlled chamber. This process significantly increases the amount of dissolved oxygen in the bloodstream, enhancing it by five to twenty times 21. Prolonged HBOT has been shown to improve mitochondrial function and reduce reactive oxygen species (ROS) levels 22. HBOT reduces ischemic brain injuries by enhancing the oxygen supply, the proliferation of neural progenitor cells within the subventricular zone (SVZ) of the hippocampus, and migrating bone marrow stem cells to the injured brain sites 23, 24. Clinical trials reveal that HBOT improves cognitive functions in Alzheimer's and Parkinson's patients 25-29 and animal models 30-35. Again, the therapeutic efficacy of HBOT on WBRT-induced cognitive deficits is still unexplored in young adult male rats model.

In this study, we first determined the cognitive function and hippocampal SGZ neurogenesis, apoptosis, phagocytosis, microglial morphology, and lipid peroxidation in young adult male rats exposed to 0 Gy and 10 Gy WBRT from 7 to 28 days post-irradiation. Additionally, we evaluated the therapeutic potential of HBOT (100% O_2_ at 2.0 atmospheres absolute [ATA] for 1 hour once a day, five days per week, and consecutively for 4 weeks after irradiation) in preventing WBRT-induced brain injury.

## Methods and Materials

### Animal studies and ethics statement

Adult male, specific pathogen-free Wistar rats (10 weeks old, weighing 380-420 g) were procured from BioLASCO Co., Ltd. and housed in groups of four at an ambient temperature of 22±1°C with a 12-hour light-dark cycle. They were provided with pellet rat chow and tap water ad libitum. All animal experiments were conducted in accordance with protocols approved by the Institutional Animal Care and Use Committee of National Cheng Kung University, Tainan, Taiwan (approved no.: 111216), according to the guidelines of the Guide for the Care and Use of Laboratory Animals published by the US National Institutes of Health, with measures taken to minimize pain and suffering. Seventy-two male Wistar rats were randomly assigned to 0 and 10 Gy irradiation groups using a randomization list method (https://www.randomizer.org/).

### WBRT model

Rats received WBRT with 0 and 10 Gy at a single time point, respectively. Assuming a value of α/β=2 Gy for brain tissue, 10 Gy represents a biologically equivalent dose of 30 Gy in 2 Gy daily fractions of conventional radiotherapy. This means the clinically relevant dose of WBRT, consistent with 30 Gy, is used for planning the target dose of the whole brain parenchyma 36-39. All anesthetized rats were irradiated using a 6 MV X-ray linear accelerator (Clinac iX, Varian Medical Systems, USA). A clinically planned treatment system was used for dosimetry, and a single dose was administrated at 3 Gy/min with a source-to-skin distance of 100 cm. Using a single open field, the head of the animal was placed at the border of the radiation field, and the whole brain was exposed to X-rays without further shielding 40. A safety margin was included to ensure complete brain irradiation and to prevent inadequate dosing due to any accidental movement during the procedure. After irradiation, the rats were kept under consistent experimental conditions to monitor their feeding and drinking behaviors, limb movements, and any local skin reactions to the radiation. Body weights were recorded on a weekly basis, and these observations were carefully documented to identify any potential changes caused by the irradiation.

### CIdU and IdU administration for tracking multiple rounds of cell division *in vivo*

To track multiple rounds of cell division *in vivo*, rats were subjected to various irradiation doses as outlined, followed by the administration of 5-chloro-2'-deoxyuridine (CIdU) one day before irradiation and continuously from day 0 to day 6 or 14. Additionally, 5-iodo-2'-deoxyuridine (IdU) was administered from day 14 to day 28 post-WBRT (CIdU at 57.5 mg/kg or IdU at 42.5 mg/kg in saline, i.p.; Sigma, St. Louis, MO, USA) to monitor the replication of cells over time. If specialized progenitor cells were maintaining the population, most cells labeled with the second analog (IdU) would have already undergone a previous round of division and would thus also be labeled with the first analog (CIdU). On the other hand, if the cell population was sustained by random cell division, most replicating cells would be labeled with either CIdU or IdU alone 41.

### Hyperbaric oxygen therapy (HBOT)

HBOT was conducted using a customized hyperbaric chamber (Chun Hung International Biomedical Technology, Tainan, Taiwan) 42, delivering 100% oxygen at 2 ATA for 1 hour per day, five days a week, for five weeks post-irradiation. The oxygen flow rate within the hyperbaric chamber was maintained at approximately 2 liters per minute, which is suitable for our chamber type and ensures consistent oxygen delivery. Compression and decompression were performed at approximately 200 kPa per minute. The HBOT sessions continuously monitored oxygen and carbon dioxide levels, ensuring oxygen levels were maintained at ≥98% and carbon dioxide levels at ≤0.03%. These protocols were well established 42-44 in our laboratory, and no seizures were observed in the animals during or after HBOT. The chamber temperature was kept between 22-25°C. We selected the protocol of daily sessions over five consecutive days per week for a total of four weeks based on evidence from previous studies demonstrating the effectiveness of such a regimen in promoting neuroplasticity and neuroprotection. Specifically, studies have indicated that repeated exposure to HBOT, administered consistently over several weeks, yields cumulative benefits in terms of reducing neuroinflammation and enhancing neurogenesis 45-47. We did not observe any complications directly related to HBOT, such as ear barotrauma, pulmonary injury, or oxygen toxicity, which are commonly reported concerns in hyperbaric treatments 48. Ear barotrauma was evaluated through behavioral observations (e.g., head shaking, balance issues), auditory startle reflex testing, and visual examination of the outer ear, none of which indicated signs of barotrauma in the treated rats. Pulmonary injury and oxygen toxicity were assessed indirectly by monitoring the overall health and well-being of the rats, with a particular focus on body weight as a general indicator of systemic health. Throughout the study period, no significant differences in body weight were observed between groups, suggesting that the HBOT regimen did not lead to pulmonary injury or oxygen toxicity.

### Neurobehavioral function

#### Radial maze assay 49

The experimental setup utilized an 8-arm radial maze, with each arm extending from a central hub. Before the training phase, the rats were allotted 5 minutes to explore the maze and access food freely. Over the subsequent five days, the animals were trained to navigate to the ends of the arms to retrieve baited food, with each training session lasting 5 minutes. Following this acclimation period, the rats were evaluated for working memory errors, defined as re-entering an arm that had already been visited, and reference memory errors, characterized by entering an arm that had never contained food. Throughout the experiment, the same four arms (numbers 2, 4, 6, and 8) were consistently baited. Each rat completed one trial per day for five consecutive days prior to surgery and one trial per week for five consecutive weeks post-surgery. The number of errors related to reference memory (long-term memory) and working memory (short-term memory) were systematically recorded 50.

#### Y maze assay

The Y-maze assay, following the methodology described by Sarter *et al.* 50, was employed to evaluate spontaneous alternation behavior, which indicates attention and working memory. The Y-maze featured three arms, each measuring 45 cm in length, 10 cm in width, and 35 cm in height, arranged at equal angles. During an 8-minute testing session, each rat was placed at the end of one arm and allowed to explore the maze freely, without the influence of external reinforcers such as food, water, or electric shock. An arm entry was defined as the rat entering an arm with all four paws. The sequence of arm entries was recorded on video for later analysis. Spontaneous alternation behavior, defined as consecutive entries into three different arms, was used to measure cognitive function. The maximum possible number of alternations was calculated by subtracting two from the total number of arm entries. The percentage of alternation behavior was then determined using the formula: (actual alternations/maximum alternations) × 100.

### Tissue preparation for histology

The chosen sampling times were based on the differentiation and maturation timeline of hippocampal neural progenitor cells, as illustrated in Figure 7, which depicts the neurogenic process, including distinct phases of proliferation, differentiation, maturation, and incorporation of new neurons into the existing hippocampal network 51. The sampling days (7, 14, and 28) were chosen to represent different phases of neurogenesis and microglial activation following radiation exposure. At each endpoint, the rats were deeply anesthetized using Zoletil (60 mg/kg; Virbac, Nice, France) and underwent transcardial perfusion with 0.9% sodium chloride (Sigma-Aldrich, St. Louis, MO, USA). The brains were carefully removed, fixed in 4% paraformaldehyde (Histolab Products AB, Sweden), and stored at 4°C for 48 hours. Subsequently, the brains were cryoprotected in a 30% sucrose solution (Sigma-Aldrich) prepared in 0.1 M phosphate buffer (pH 7.4) before being processed for paraffin embedding and sectioning.

### TUNEL assay for apoptotic cells

In situ apoptosis detection kits (Immunotech Corporation, USA) was utilized to assess the apoptosis rate using the terminal deoxynucleotidyl transferase (TdT)-mediated dUTP nick end-labeling (TUNEL) method. TUNEL-positive cells were counted under a microscope for quantitative analysis. The apoptotic rate was calculated as the percentage of labeled cells within six fields of the dentate gyrus in the hippocampus. The staining results were analyzed and evaluated using Image-Pro Plus software (Media Cybernetics, USA).

### Histological examination and immunohistochemical staining

Rat brains were extracted and fixed in formalin overnight, followed by embedding in paraffin. Brain sections from each experimental group were cut coronally at 5 μm thickness using a microtome. These sections were then stained using hematoxylin and eosin (H&E) and analyzed under a light microscope (Carl Zeiss Microscopy GmbH, Jena, Germany) for histopathological assessment. Damage was quantified by evaluating the extent of the lesion area and morphological alterations. Lesion severity was scored on a scale from 0 (no pathological changes) to 4 (lesions covering 75%-100% of the field) 52. Morphological changes were rated from 0 (normal morphology) to 3 (severe structural disorganization and inflammation) 53. These scores were multiplied to derive an overall damage score for each section, and the average was calculated across four areas per dentate gyrus region.

For the assessment of microglial activation, immunohistochemical staining was conducted using an anti-Iba-1 rabbit antibody (GeneTex Inc., San Antonio, Texas, USA) applied at 4°C overnight. Following this, the sections were washed with PBS and incubated with N-Histofine Simple Stain MAX PO (Nichirei Biosciences Inc., Tokyo, Japan) as a secondary antibody at room temperature for one hour. The primary antibody binding was developed using 3-amino-9-ethylcarbazole (AEC) in acetate buffer (BioGenex, San Ramon, CA, USA). Building on the methods outlined by Resende *et al.* 54, we measured the soma size of microglia by tracing around each cell body with a contour tool under a 100x oil immersion objective and analyzing the images with Zen Software (Carl Zeiss). The mean area of microglial cell bodies was calculated specifically for the dentate gyrus, summarizing the measurements across 10 immunostained sections per dentate gyrus region for each rat.

### Immunofluorescence staining for Neural Stem Cells (NSCs), neuroblasts, and neuronal proliferation and differentiation

The previously prepared hippocampal slides were initially incubated in 2 mol/L HCl for 30 minutes, followed by a rinse in 0.1 mol/L boric acid (pH 8.5) for 3 minutes at room temperature. The slides were then incubated overnight at 4°C with primary antibodies diluted in PBS containing 0.5% normal bovine serum. Following this, secondary antibodies were applied for 1 hour at room temperature. The primary antibodies utilized included IdU (1:400, Chemicon), CldU (anti-BrdU, 1:400, Accurate), doublecortin (DCX, 1:200, Cell Signaling), nestin (1:1,000, Sigma), ionized calcium-binding adaptor molecule 1 (Iba1, 1:1,000, Dako), and neuronal nuclei antigen (NeuN, 1:1,000, Chemicon). Visualization of the cells was achieved using Alexa Fluor 488-conjugated goat anti-rabbit IgG (1:400, Invitrogen, CA, USA, #A11008. RRID: AB_143165) and Alexa Fluor 568-conjugated goat anti-rabbit IgG (1:400, Invitrogen, #A11008. RRID: AB_2534072), with excitation and emission wavelengths of 495/525 nm and 578/603 nm, respectively. The sections were subsequently mounted with a fluorescent medium (Agilent Technologies, Glostrup, Denmark), and fluorescence signals were captured using an upright fluorescence microscope (Carl Zeiss, Jena, Germany). Images were acquired with a digital camera connected to a computer equipped with Axioscope version 4 software (Carl Zeiss). To confirm specificity, negative control sections were processed without the addition of primary antibodies. A pathologist counted triple or quadruple co-labeled cells—including CldU/IdU/DAPI, CldU/IdU/TUNEL/DAPI, Iba1/Nestin/CldU/TUNEL, Iba1/DCX/CldU/TUNEL, and Iba1/NeuN/CldU/TUNEL—across 30 frames in five sections (×200 magnification). All cell counts were performed in a blinded manner to eliminate bias.

### Detection of Lipid Hydroperoxides (LPO)

The formation of lipid hydroperoxides was assessed using a commercially available lipid hydroperoxide assay kit (#ab133085, Abcam, Waltham, MA, USA). Samples were first centrifuged at 1,500 g for 5 minutes at 0°C. Following this, 500 µl of the bottom chloroform layer was collected and combined with a chloroform-methanol solvent. Then, 50 µl of chromagen was added to the sample mixtures, which were incubated at room temperature for 5 minutes. The absorbance was subsequently measured at 500 nm using a 96-well plate.

### Determination of Malondialdehyde (MDA)

For measuring tissue levels of MDA, we used the supernatants after homogenization and an MDA assay kit (#ab118970, Abcam, Waltham, MA, USA). Briefly, 200 μg of proteins were mixed with thiobarbituric acid (TBA) and heated for 1 hour at 95^o^C. After cooling on ice, the absorbance of the samples was measured at 532 nm, and a standard concentration curve was used to calculate the MDA content.

### Statistical analysis

No outliers were excluded from the statistical analysis. The analysts were blinded to the conditions of any experiment where data points were manually obtained. Statistical analyses were conducted using GraphPad Prism 7.01 (GraphPad Software Inc., CA, US). Behavioral performance data were analyzed using two-way ANOVA with Tukey's multiple comparisons tests. Histological staining and immunostaining data were analyzed using the Kruskal-Wallis and Dunn's post hoc tests. Data were expressed as mean ± SD. P-values < 0.05 were considered statistically significant.

## Results

### Effects of WBRT on hippocampal cellular morphology and microglial activity

**Figure 1:**
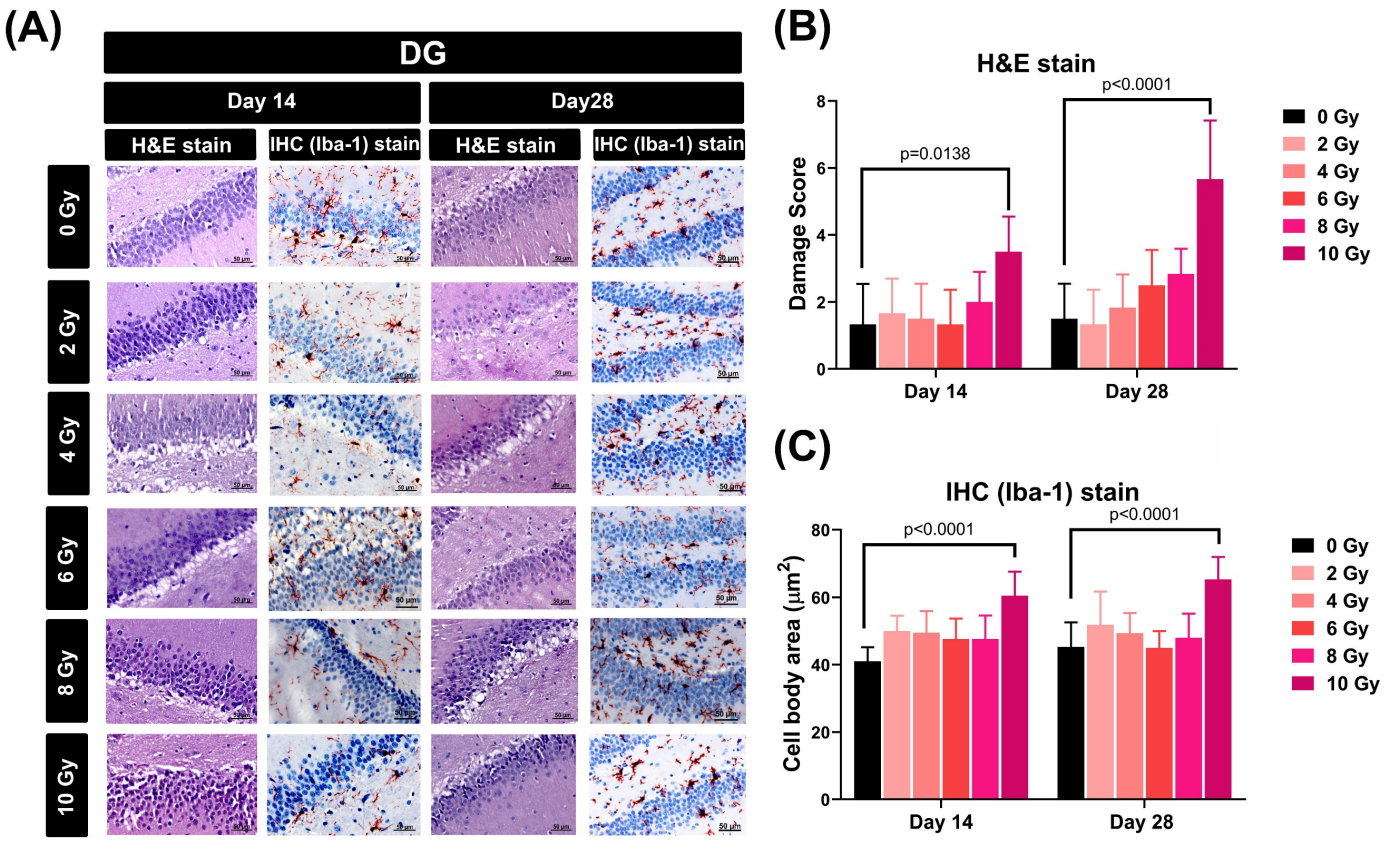
Effects of different doses of WBRT on cellular morphology and microglial activity within the dentate gyrus (DG) region of rats assessed by H&E and IHC staining. **(A)** Representative images of the DG of rat hippocampi stained with H&E and IHC for Iba-1 at 14 and 28 days post-WBRT with increasing radiation doses (0, 2, 4, 6, 8, and 10 Gy). Scale bars represent 50 µm. **(B)** Quantitative analysis of damage scores in the DG, assessed by H&E staining, at days 14 and 28 post-WBRT. The scores represent the degree of pathological changes with increasing WBRT doses, showing a significant increase in tissue damage at 10 Gy. **(C)** Microglial cell body size in the DG, quantified by the cell body area stained for Iba-1, at 14 and 28 days post-WBRT. WBRT at 10 Gy, but not other doses below 10 Gy, caused a significant microglial swelling (p<0.0001). Bars represent the means±SD of n=6 for each group of rats.

To assess the impact of varying doses of WBRT on cellular morphology and microglial activity within the dentate gyrus (DG) of the hippocampus, both hematoxylin and eosin (H&E) and immunohistochemical (IHC) staining techniques were employed to detect cellular damage score and microglia dysmorphology, respectively (**Figure 1A**). The results showed that when evaluated at 14 and 28 days post-WBRT, exposure to 10 Gy, but not other doses, caused a significant increase in both the cellular damage score (**Figure 1B**) and the extent of microglial swelling (**Figure 1C**).

### Effects of HBOT on the WBRT-induced spatial learning and memory deficits evaluated on Day 28 post-WBRT

**Figure 2:**
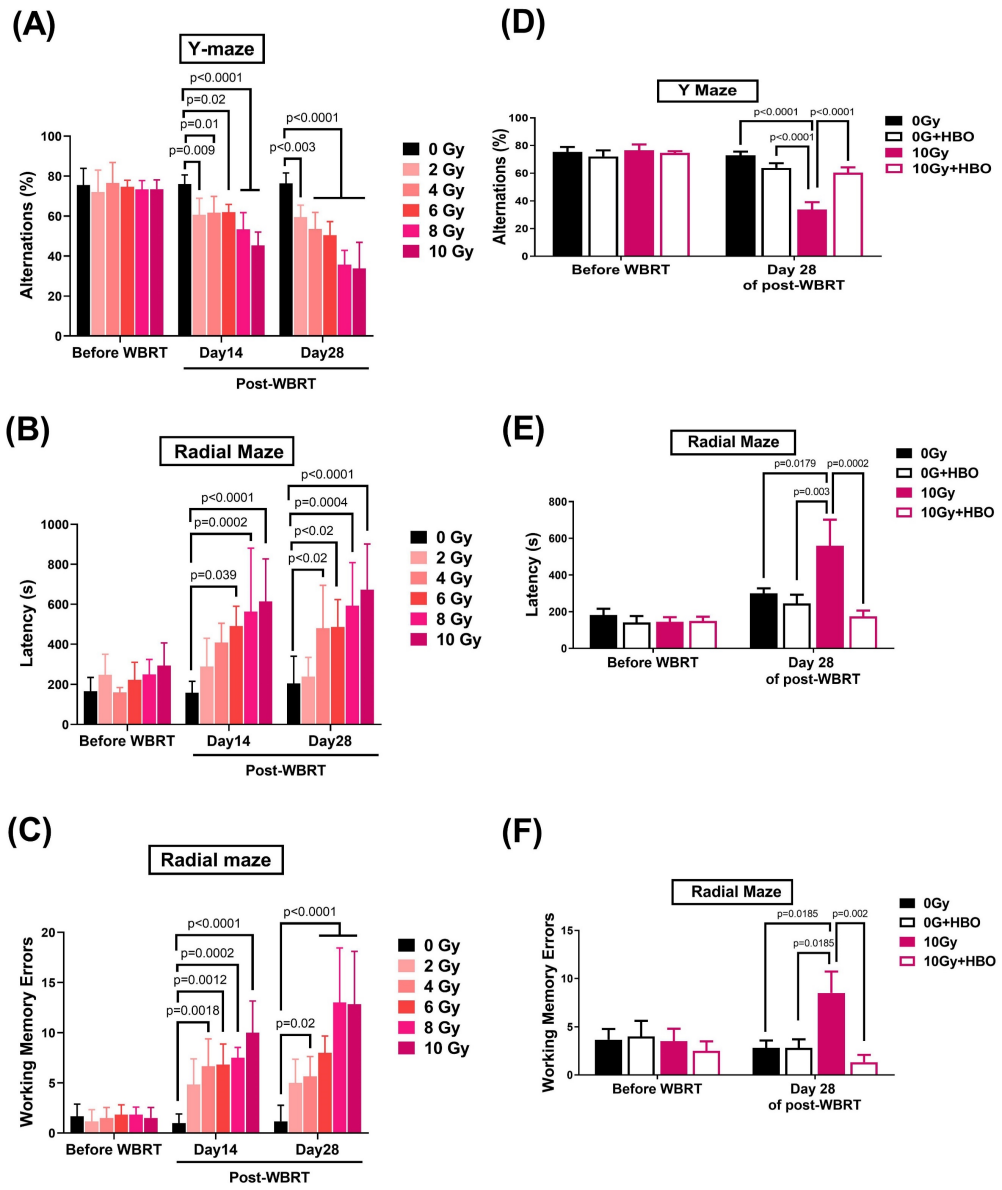
Effects of hyperbaric oxygen therapy (HBOT) on whole brain radiotherapy (WBRT)-induced spatial learning and memory deficits in rats evaluated at 28 days post-WBRT. The dose responses from 0 Gy to 10 Gy of WBRT on **(A)** spontaneous Y maze alternation for spatial working memory assessment. **(B)** and** (C)** The 8-arm Radial maze was used to measure spatial learning and memory in rats with various doses of radiation exposure. To evaluate the HBOT on neurobehavioral function, both **(D)** Y-maze and **(E &F)** Radial-maze were evaluated before and 28 days after a WBRT (10 Gy). Bars represent the means±SD of n=6 for each group. 0 Gy+NBA: rats treated with 0 Gy WBRT and normobaric atmosphere. 0 GY WBRT+HBOT: Rats received 0 Gy WBRT and HBOT. 10 Gy WBRT+NBA: Rats received 10 Gy WBRT and normobaric atmosphere. 10 Gy WBRT+HBOT: rats received 10 Gy WBRT and HBOT. Bars represent the means±SD of n=6 for each group of rats.

Y-maze (**Figure 2A**) and radial maze (**Figure 2B & C**) assessments were performed on days 14 and 28 following WBRT at doses from 0 to 10 Gy to evaluate the impact of varying radiation exposure levels on spatial learning and memory capabilities. The results indicated a dose-dependent significant decline in alternation performance in the Y-maze (**Figure 2A**) starting at 2 Gy, accompanied by increased latency during exploration (**Figure 2B**) and heightened working memory errors in the radial maze (**Figure 2C**), with these effects being particularly pronounced at 10 Gy WBRT. Observations on day 28 mirrored those recorded earlier, leading to the selection of a 10 Gy WBRT dose for subsequent experiments.

Four groups of rats, including 0 Gy WBRT group, 0 Gy WBRT+HBO group, 10 Gy WBRT+NBA group, and 10G WBRT+HBOT group, were subjected to Ymaze and dial maze tests to assess their spatial learning and memory function and working memory function. Two days before WBRT started, there was an insignificant maze test. However, when evaluated on Day 28 post-WBRT, the 10 Gy WBRT rats treated with normobaric oxygen displayed spatial learning and memory loss. Compared to the 0 Gy WBRT group rats, the 10 Gy WBRT group rats had a significantly lower % of alteration in the Y maze test (**Figure 2D**) as well as higher values of both the latency and working memory errors in radial maze tests (**Figure 2E** & **F**). Compared to the 10 Gy WBRT+NBA group rats, the 10 Gy WBRT+HBOT group rats had significantly higher values of % alterations in Y maze trials and significantly lower values of both the latency (%) and working memory errors (times) in radial maze trials (**Figure 2**). To monitor the potential systemic effects of WBRT and HBOT, the body weights of the rats were recorded weekly. Despite the known stress induced by WBRT, there were no significant changes in body weight among the groups throughout the study period, including the HBOT-only group (p > 0.05, data not shown). The maintenance of body weight across all groups, including the 0 Gy + HBOT group, suggests that neither WBRT nor HBOT led to gross physiological changes in body mass.

### Effects of HBOT on the WBRT-induced apoptosis of newly-born NSC and neuroblasts and microglial activation on day 7 post-WBRT

**Figure 3:**
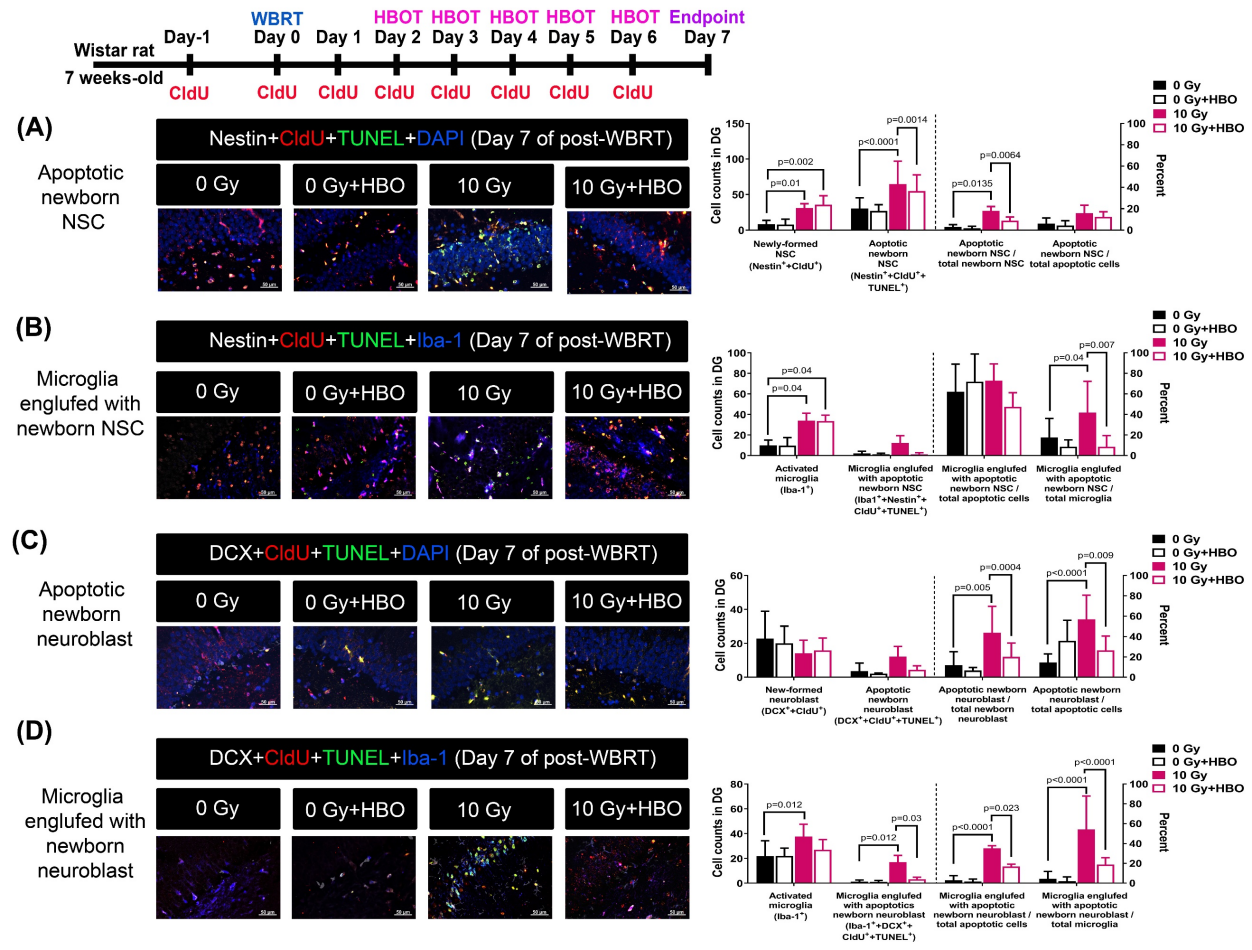
HBOT attenuated the WBRT-induced SGZ neurogenesis deficits and apoptosis in the subacute phase (day 7). **(A)** Representative examples of apoptotic newborn neural stem cells (NSC) in the young adult rat SGZ in each group. Quantification of the effects of 0 Gy- and 10 Gy-irradiated with or without HBOT at day 7 after WBRT, on the newborn NSC survival (nestin^+^+CldU^+^) and apoptotic newborn NSC (nestin^+^+CldU^+^+TUNEL^+^). **(B)** Representative examples of apoptotic newborn NSC engulfed by microglia in the young adult rat SGZ in each group. Quantification of the effects of 0 Gy- and 10 Gy-irradiated with or without HBOT at day 7 after WBRT, on the active microglia (Iba-1^+^) and apoptotic newborn NSC engulfed by microglia (nestin^+^+CldU^+^+TUNEL^+^+Iba-l^+^). **(C)** Representative examples of apoptotic newborn neuroblast in the young adult rat SGZ in each group. Quantification of the effects of 0 Gy- and 10 Gy-irradiated with or without HBOT at day 7 after WBRT, on the newborn neuroblast survival (DCX^+^+CldU^+^) and apoptotic newborn neuroblast (DCX^+^+CldU^+^+TUNEL^+^). **(D)** Representative examples of apoptotic newborn neuroblasts engulfed by microglia were found in the adult rat SGZ in each group. Quantification of the effects of 0 Gy- and 10 Gy-irradiated with or without HBOT at day 7 after WBRT, on the active microglia (Iba-1^+^) and apoptotic newborn neuroblast engulfed by microglia (DCX^+^+CldU^+^+TUNEL^+^+Iba-l^+^). Bars represent the means±SD of n=6 for each group of rats. Please see the explanations for the abbreviation of different group rats in the legends of Fig. 2.

In the subacute stage (day 7) after WBRT, we also found a similar phenomenon in the cell population (increase in newborn NSC and neuroblasts), cell death (increase in apoptosis of newborn NSC and neuroblasts), and microglia activation (engulfment of newborn NSC and neuroblasts), which were significantly attenuated by HBO therapy (**Figure 3**).

### Effects of HBOT on the WBRT-induced SGZ neurogenesis deficits and apoptosis of neurons during the chronic phase (day 28 post-WBRT)

**Figure 4:**
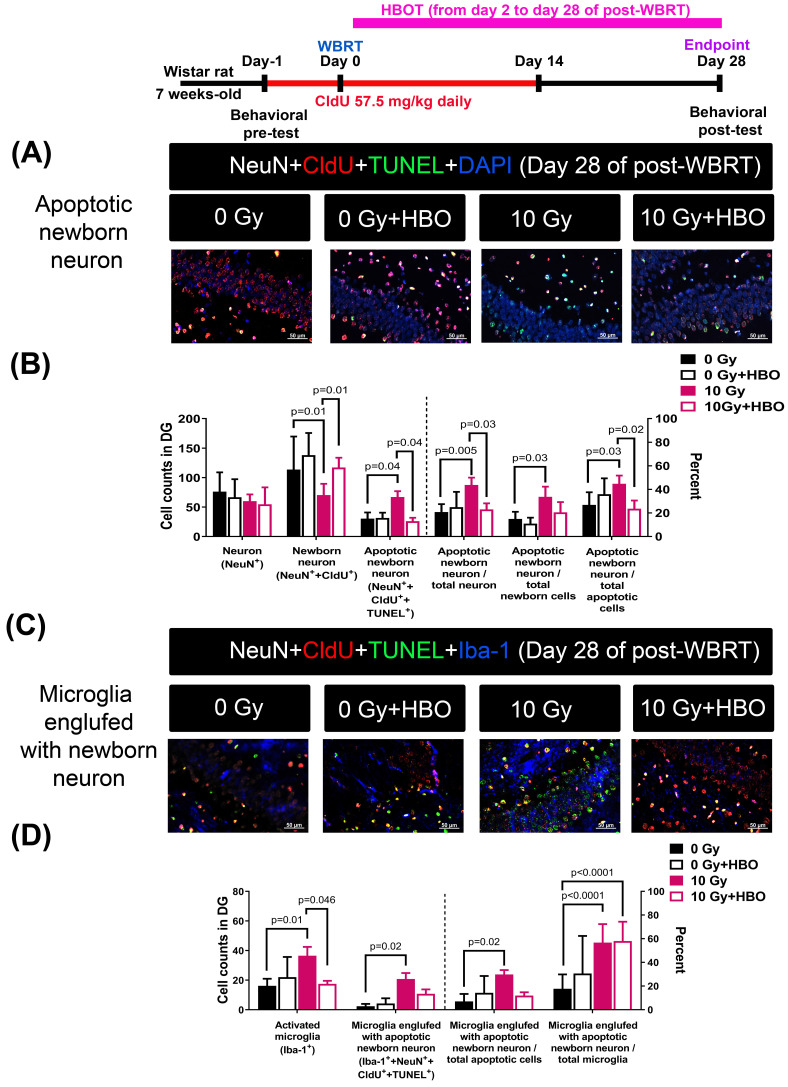
Effects of HBOT on the WBRT-induced altered counts of apoptotic newborn neurons as well as microglia engulfed with apoptotic neurons in the 0 Gy at 28 days post-WBRT for different groups of rats. Bars represent the means±SD of n=6 for each group of rats. Please see the explanations for the abbreviation of different group rats in the legends of Fig. 2.

**Figure 4A** and **Figure 4B** revealed that compared to the 0 Gy WBRT group rats, the 10 Gy WBRT group rats had significantly lower values of newborn neurons (P=0.01) but significantly higher values of apoptotic newborn neurons (P=0.04), apoptotic newborn neurons/total neurons (P=0.005) and apoptotic newborn neurons/total newborn neurons (P=0.03). Compared to the 10 Gy WBRT group rats, the 10 Gy WBRT+HBOT group rats had significantly higher values of newborn neurons (P=0.01) and lower values of apoptotic newborn neurons (p=0.04).

**Figure 4C** and **Figure 4D** showed that compared to 0 Gy WBRT group rats, the 10 Gy WBRT group rats had significantly higher values of microglial number (P=0.01), apoptotic newborn neurons engulfed by microglia (P=0.02), apoptotic newborn neurons engulfed by microglia/total apoptotic neurons (P=0.02) and apoptotic newborn engulfed by microglia/total microglia (P<0.0001). Compared to the 10 Gy WBRT group rats, the 10 Gy WBRT+HBOT group mice had significantly lower values of microglial numbers (P=0.046).

### Effects of HBOT on the WBRT-induced altered numbers of proliferative cells (CldU^+^ cells or IdU+ cells) or serial replicating cells (or CldU^+^ IdU^+^ co-labeled cells) evaluated on Day 28 post-WBRT

**Figure 5:**
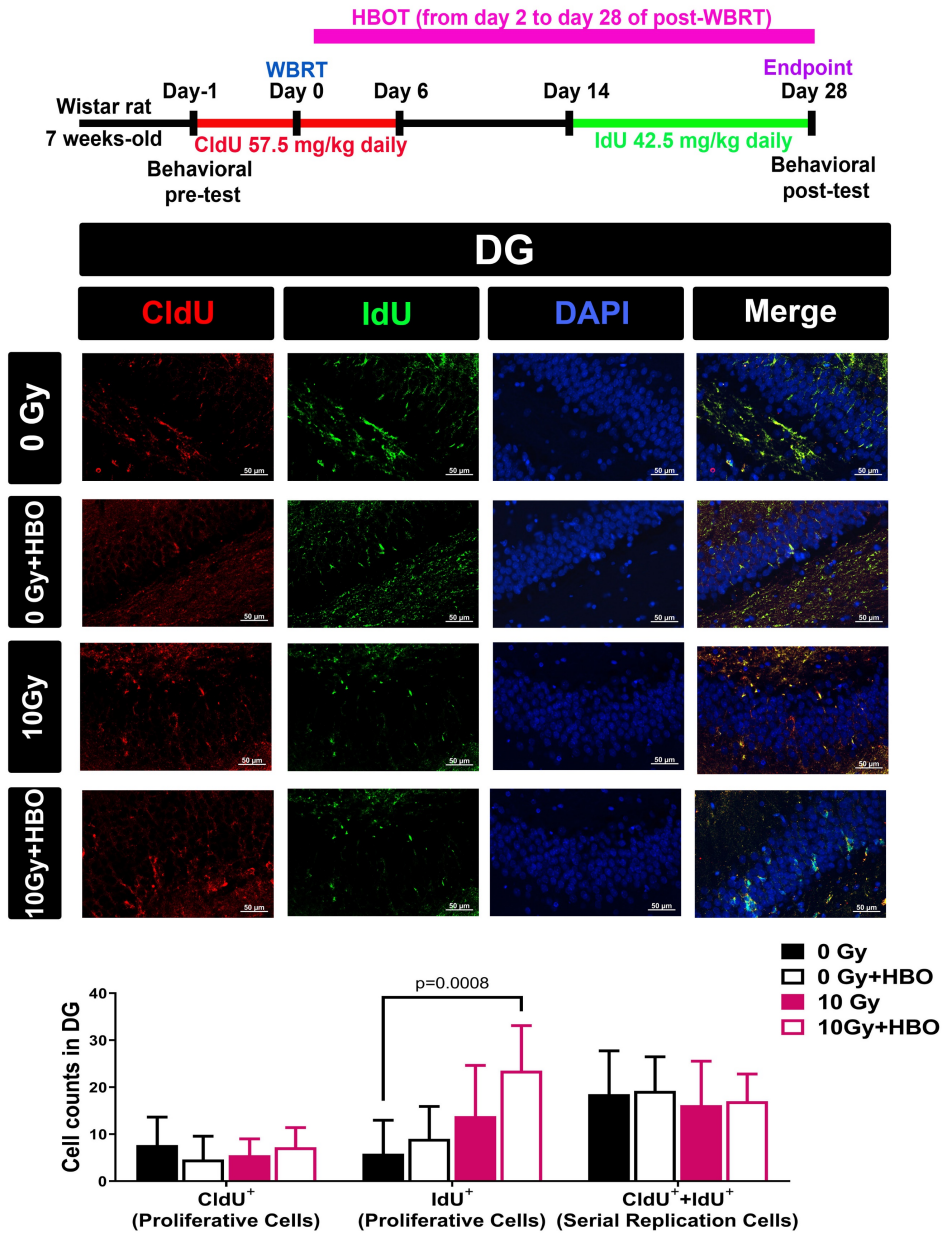
Effects of HBOT on the WBRT-induced altered counts of survival cells, proliferative cells, and serial replicating cells at day 28 post-WBRT for different groups of rats. To determine the serial replication of specialized progenitors, rats received two different thymidine analogs, namely CldU (consecutive administration 7 days after WBRT) and IdU (consecutive administration from day 14 to day 28 after WBRT), to detect the serial replicating cells (CldU-IdU-co-labeled cells). Upper: Representative examples of the survival, proliferation, and serial replicating cells (C1dU^+^+IdU^+^) in the DG in 0 Gy-, or 10 Gy-irradiated rats at day 28 post-WBRT. Lower: Bars represent the means±SD of n=6 for each group. Please see the explanations for the abbreviations of different group rats in the legends of Fig. 2.

To determine the numbers of newborn proliferative cells and serially replicating cells in DG, rats received two different thymidine analogs, namely CldU (one day before WBRT and consecutive 7 days post-WBRT) and IdU (consecutive Day 14 to Day 28 post-WBRT). Compared to the 0 Gy WBRT group rats, the 10 Gy WBRT group rats had an insignificant difference in the numbers of newborn proliferative cells or serial replicating cells in the DG of the hippocampus (**Figure 5**). Again, compared to the 10 Gy group rats, the 10 Gy+HBOT group rats had an increase of newborn proliferative cells (IdU^+^) but an insignificant difference in the numbers of serially replicating cells (**Figure 5**).

### Effects of HBOT on the WBRT-induced lipid peroxidation in the hippocampal homogenates evaluated at Day 28 post-WBRT

**Figure 6:**
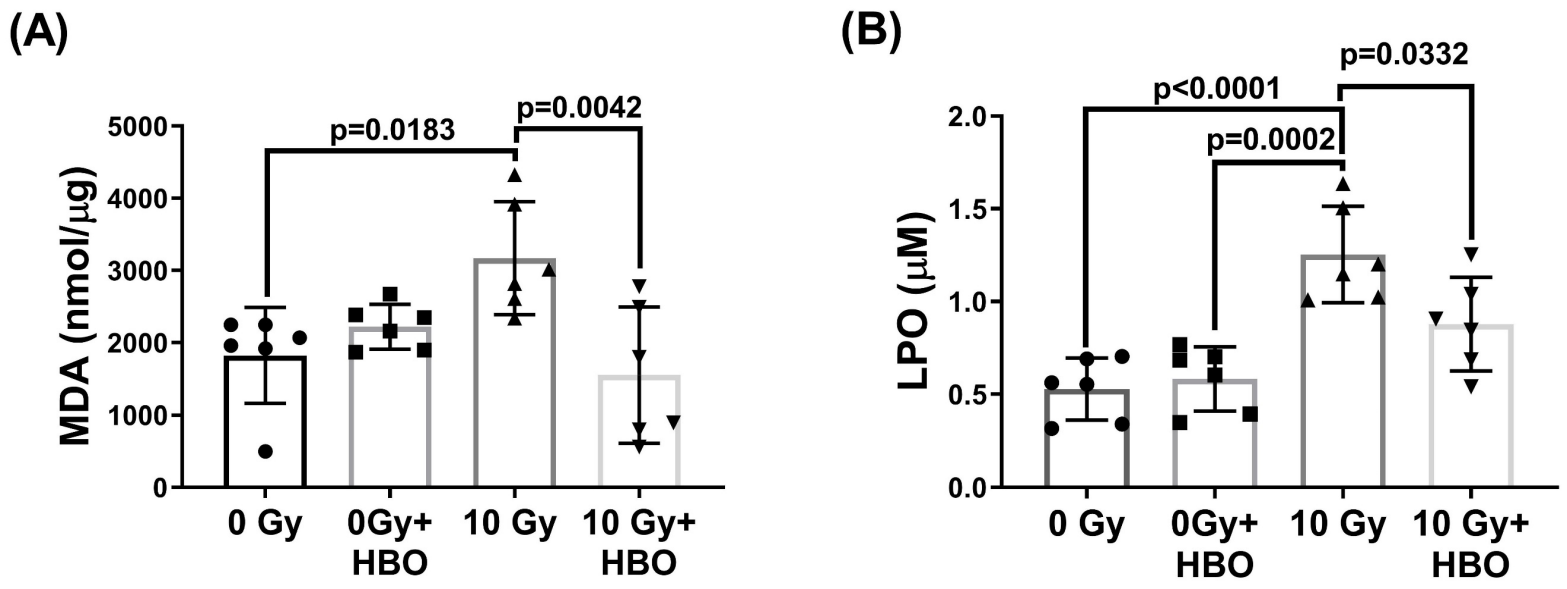
Effects of HBOT on the WBRT-induced altered contents of both MDA and LPO in the DG of different groups of rats at Day 28 post-WBRT. Bars represent the means±SD of n=6 for each group. Please see the explanations for abbreviations of different group rats in the legends of Fig. 2.

Compared to the 0 Gy WBRT group rats at day 28 post-WBRT, the 10 Gy WBRT group rats had significantly higher values of MDA (P=0.0183) and LPO (P<0.0001)** (Figure 6)**. However, compared to the 10 Gy group, the 10 Gy +HBOT groups had significantly lower values of MDA (P=0.0042) and LPO (P=0.0332).

## Discussion

Patients who received WBRT displayed hippocampal injury accompanied by cognitive impairment 36, 55, 56. Previous studies on mice exposed to 1 or 10 Gy WBRT reported significant structural alterations in hippocampal neurons in the dentate gyrus by day 30 post-exposure 57, 58. Similarly, fractionated WBRT at 20 Gy in rats led to histopathological changes in the neurogenic region, which were linked to cognitive impairment 59. *Ex vivo* studies have also shown that 10 Gy irradiation inhibits long-term potentiation in cultured hippocampal slices, resulting in functional impairments in the dentate gyrus 60. Furthermore, WBRT with 10 Gy has been linked to age-dependent impairments in cortical synaptic plasticity 61. Collectively, radiation-induced cognitive deficits can arise from vascular damage, neuroinflammation, impairment of neurogenesis, and disrupted neuronal function 62. Our present studies provide new evidence that WBRT with 10 Gy on 28 days leads to cognitive deficits via inhibition of neurogenesis, increased microgliosis, newborn neuron apoptosis, and elevated lipid peroxidation in the dentate gyrus of the hippocampus in adult male rats.

Our findings suggest a threshold effect where significant histological damage is evident only at higher doses (e.g., 10 Gy). In contrast, lower doses (e.g., 2-4 Gy) primarily induce functional changes, resulting in behavioral impairments without detectable cellular damage or microglial activation within the timeframe of our study. Behavioral assays, such as the Y-maze and radial maze, are highly sensitive to even minor disruptions in synaptic plasticity and hippocampal network integrity. Therefore, radiation exposure at lower doses (e.g., 2-4 Gy) could impair spatial learning and memory, leading to behavioral changes without significant histological damage or increased microglial activation during the acute observation period. These functional deficits likely result from early synaptic connectivity and neurotransmission disturbances rather than overt neuronal death or inflammation. In contrast, significant histological changes, such as increased damage scores and microglial swelling, were evident only at higher doses (e.g., 10 Gy), which exceeded the compensatory repair mechanisms of the brain. This dose-dependent effect highlights a threshold where low-dose radiation exposure can lead to subtle, cumulative disruptions in neuronal function, while higher doses trigger extensive cellular damage and inflammation. This finding aligns with previous studies suggesting that low doses of radiation may not elicit significant short-term effects but can lead to subtle and cumulative alterations over a longer duration 63, 64.

The role of adult neurogenesis in cognitive processes in rodents remains a topic of ongoing debate 65. Our findings indicate that while mature differentiated neurons remain largely unaffected by irradiation, cognitive impairments are primarily due to the loss of newly-born neurons in the hippocampus, which is linked to increased lipid peroxidation. This suggests that disruptions in adult neurogenesis are a key mechanism underlying radiation-induced cognitive deficits. HBOT has been shown to increase blood oxygen, improve mitochondrial activity, and reduce tissue radical oxygen species levels 21. It also enhances recovery from ischemic brain injury by promoting the proliferation of neural progenitor cells in the subventricular zone and facilitating the migration of bone marrow-derived stem cells to injured areas 23, 24. Previous studies have demonstrated the effectiveness of HBOT in enhancing cognitive functions in patients with neurodegenerative diseases, such as Alzheimer's and Parkinson's disease 25-29, as well as in animal models 27, 28, 31-33. Consistent with these findings, our current study shows that HBOT alleviates cognitive deficits in rats subjected to WBRT by increasing the proliferation of newly-born neurons and reducing microgliosis and lipid peroxidation within the subgranular zone of the hippocampal dentate gyrus. These results align with prior studies showing that interventions like indomethacin can partially restore neurogenesis following brain irradiation in rats 66. Similarly, antioxidants such as alpha-tocopherol improve global cognition in patients with temporal lobe radionecrosis following radiation therapy for nasopharyngeal carcinoma 67. Additionally, alpha-lipoic acid has been reported to attenuate radiation-induced brain damage by reducing oxidative stress in mice 68. Interestingly, some studies have shown that irradiation can enhance hippocampus-dependent cognition in mice deficient in extracellular superoxide dismutase 69, suggesting that oxidative stress modulation plays a complex role in neurogenesis and cognitive outcomes. Future studies should focus on elucidating the precise mechanisms through which HBOT reduces oxidative stress and lipid peroxidation to support neurogenesis, and how these changes contribute to long-term improvements in cognitive function.

As resident immune cells in the central nervous system, microglia play a crucial role in neuroinflammatory responses and reactive oxygen species production 70. In response to radiation injury, generally, active microglia become more mobile and phagocytotic. Although morphological changes such as increased cell body size were not evident in our current study, we observed increased microglial numbers and phagocytic activity in the dentate gyrus following WBRT. HBOT significantly decreased microglial proliferation without reducing their phagocytic capacity, suggesting that HBOT exerts its anti-inflammatory effects primarily by modulating microglial recruitment rather than impairing their functional activity. This observation suggests a nuanced effect of HBOT on microglial dynamics. Specifically, HBOT appears to reduce the proliferation or recruitment of microglia without inhibiting their functional role in phagocytosis. The remaining microglia may be sufficient to maintain the necessary clearance of apoptotic cells and debris, even with reduced inflammation 13, 71. Microglial phagocytosis is critical for supporting neurogenesis by creating an environment conducive to the proliferation and differentiation of neural progenitor cells 13. The fact that HBOT decreases microglial numbers while preserving their phagocytic activity is crucial. This balance allows cellular debris clearance without triggering excessive inflammatory responses, which could otherwise disrupt neurogenesis 72.

Additionally, our data demonstrated that WBRT reduced the number of newborn neurons in the subgranular zone (SGZ) of the hippocampus, while the number of mature neurons remained unaffected at day 28 post-WBRT. These findings suggest that neurons born within 2-4 weeks after radiation exposure are particularly vulnerable, whereas younger neural progenitors may exhibit greater resilience. Our results are consistent with findings from Mineyeva *et al.* 73, who reported that radiation suppresses the division of hippocampal stem cells, which is only partially alleviated in the long term. Although we observed that radiation did not affect the number of proliferating cells and serially replicating cells in the SGZ, our study suggests that radiation has a detrimental impact on the maturation and survival of newly formed neurons, contributing to impaired neurogenesis.

The present study has some limitations. First, the findings are based on an acute observation period of 28 days following WBRT. The long-term effects of WBRT and HBOT on neurogenesis and cognitive function require further investigation to better understand the potential for chronic radiation-induced damage and the sustained benefits of HBOT. Second, the HBOT regimen used in this study involved a single session per day, selected to minimize the risks of oxygen toxicity, such as oxygen-induced seizures or pulmonary complications. However, it is important to acknowledge that more aggressive or alternative HBOT regimens, such as different pressure levels or multiple daily sessions, might yield different outcomes. The variability in sensitivity to oxygen toxicity among different species further complicates this aspect 74 75. Future research should explore alternative HBOT protocols to determine the most effective and safe regimen for radiation-induced brain injury. Third, the sample size of each experimental group was relatively small, which may limit the statistical power of the findings. Future studies with larger sample sizes will be necessary to confirm these results and improve the robustness of the conclusions drawn. Fourth, this study was conducted exclusively on adult male Wistar rats, which poses limitations regarding the generalizability of the results to other species, including humans. Furthermore, female animals were not included in this study, which restricts our understanding of sex-specific differences in response to radiation and HBOT. Considering potential sex-based variations in hormonal influences on radiation sensitivity and neuroinflammation, future studies should incorporate both male and female subjects. Lastly, our behavioral assessments focused primarily on spatial learning and memory, which are closely associated with hippocampal function. While these tests are valuable for understanding hippocampal-dependent cognitive impairments, additional behavioral evaluations encompassing other domains, such as anxiety, motor function, and social behavior, would help provide a more complete assessment of the cognitive and functional impacts of WBRT and the effects of HBOT. By addressing these limitations, future studies will be able to better elucidate the therapeutic potential of HBOT for mitigating radiation-induced cognitive deficits and provide more comprehensive data for eventual translation to clinical practice.

## Conclusions

**Figure 7:**
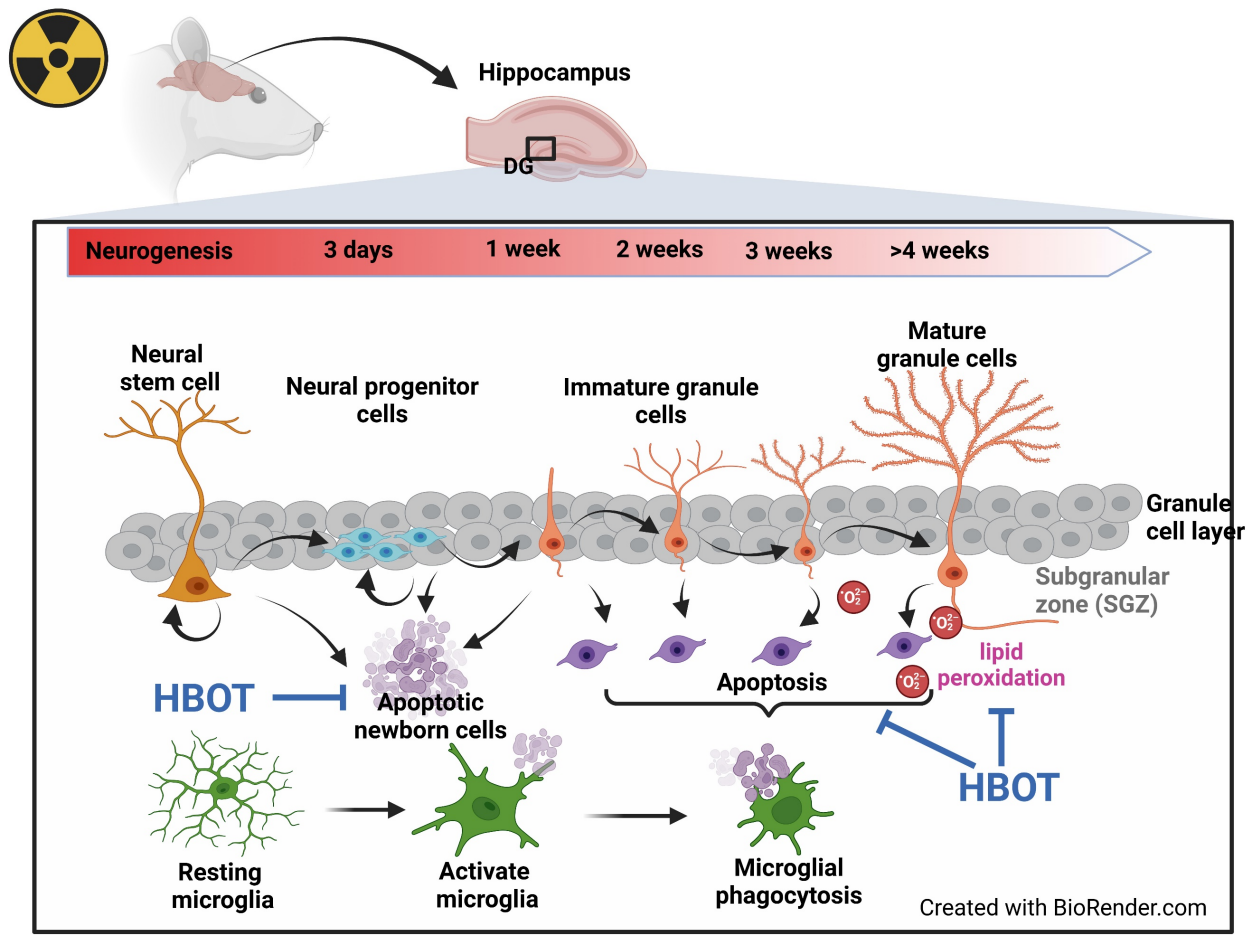
Data summary. Seven days post-WBRT, there was a notable increase in hippocampal neurogenesis in the dentate gyrus (DG), although this was accompanied by significant apoptosis. Changes in the cellular composition of the DG were evident on days 7 and 28 post-WBRT, characterized by an increase in neuroblast and neuron numbers, with a significant proportion undergoing apoptosis linked to microglial phagocytosis, which correlated with cognitive decline. Additionally, lipid peroxidation levels significantly increased by day 28 post-WBRT. HBOT improved cognitive functions by reducing WBRT-induced lipid oxidation, newly formed cell apoptosis, and microglia phagocytosis.

Our findings indicate that WBRT may inhibit the generation of new neurons while simultaneously enhancing microglial activation, apoptosis of newborn neurons, and lipid peroxidation in the dentate gyrus of the rat brain. These pathological changes likely contribute to the observed cognitive deficits, neuronal death, and impaired neurogenesis in the hippocampus. Conversely, HBOT effectively counters these adverse effects, significantly reducing cognitive impairments, neuronal loss, and disruptions in hippocampal neurogenesis induced by WBRT in adult male rats (as summary in **Figure 7**). These results underscore the potential of HBOT as a therapeutic strategy to mitigate the neurodegenerative impacts of brain irradiation.
